# A novel method for RNA extraction from FFPE samples reveals significant differences in biomarker expression between orthotopic and subcutaneous pancreatic cancer patient-derived xenografts

**DOI:** 10.18632/oncotarget.11809

**Published:** 2016-09-01

**Authors:** Malachia Hoover, Yvess Adamian, Mark Brown, Ali Maawy, Alexander Chang, Jacqueline Lee, Armen Gharibi, Matthew H Katz, Jason Fleming, Robert M Hoffman, Michael Bouvet, Robert Doebler, Jonathan A Kelber

**Affiliations:** ^1^ Department of Biology, California State University Northridge, Northridge, CA, USA; ^2^ Claremont BioSolutions, Upland, CA, USA; ^3^ UCSD Moores Cancer Center and Department of Surgery, La Jolla, CA, USA; ^4^ Department of Surgery, M.D. Anderson Cancer Center, Houston, TX, USA; ^5^ AntiCancer Inc., San Diego, CA, USA

**Keywords:** FFPE RNA extraction, microHomogenizer™, pancreatic cancer, patient-derived orthotopic xenografts (PDOX) tumor microenvironment, cancer biomarkers

## Abstract

Next-generation sequencing (NGS) can identify and validate new biomarkers of cancer onset, progression and therapy resistance. Substantial archives of formalin-fixed, paraffin-embedded (FFPE) cancer samples from patients represent a rich resource for linking molecular signatures to clinical data. However, performing NGS on FFPE samples is limited by poor RNA purification methods. To address this hurdle, we developed an improved methodology for extracting high-quality RNA from FFPE samples. By briefly integrating a newly-designed micro-homogenizing (mH) tool with commercially available FFPE RNA extraction protocols, RNA recovery is increased by approximately 3-fold while maintaining standard A260/A280 ratios and RNA quality index (RQI) values. Furthermore, we demonstrate that the mH-purified FFPE RNAs are longer and of higher integrity. Previous studies have suggested that pancreatic ductal adenocarcinoma (PDAC) gene expression signatures vary significantly under *in vitro* versus *in vivo* and *in vivo* subcutaneous versus orthotopic conditions. By using our improved mH-based method, we were able to preserve established expression patterns of KRas-dependency genes within these three unique microenvironments. Finally, expression analysis of novel biomarkers in KRas mutant PDAC samples revealed that PEAK1 decreases and MST1R increases by over 100-fold in orthotopic versus subcutaneous microenvironments. Interestingly, however, only PEAK1 levels remain elevated in orthotopically grown KRas wild-type PDAC cells. These results demonstrate the critical nature of the orthotopic tumor microenvironment when evaluating the clinical relevance of new biomarkers in cells or patient-derived samples. Furthermore, this new mH-based FFPE RNA extraction method has the potential to enhance and expand future FFPE-RNA-NGS cancer biomarker studies.

## INTRODUCTION

Next-Generation Sequencing (NGS), such as RNA-seq or whole-genome analysis, of patient-derived tumor tissue in combination with state-of-the art bioinformatics holds great potential to improve disease outcomes by identifying novel biomarkers of cancer onset, progression and therapy resistance [1–3]. Among the advantages of NGS over other technologies is the ability to perform high-resolution transcriptome monitoring to identify isoform variations, outlier expression patterns and expression signatures of low expressing genes or long noncoding RNAs [4]. Importantly, these data can help to differentiate between unique molecular subtypes of various malignancies for the purpose of guiding diagnosis, prognosis and therapeutic interventions [5, 6].

To succeed in this endeavor, it is essential to correlate clinical and NGS data from a wide sampling of patient tumors that take into account a physiological tumor microenvironment. However, understanding the effects of the tumor microenvironment on gene expression and, in particular, cancer-cell biomarker profiles is hindered by the fact that researchers have limited access to fresh tumor tissue, which harbor good-quality and easily-accessible nucleic acid material - these samples are not routinely obtained in hospitals due to the logistical challenges of collecting, processing and banking fresh-frozen tissue [7]. Formalin-fixed paraffin-embedded (FFPE) samples, however, are routinely collected for pathology analysis, and substantial archives of FFPE tissues from all types of malignancies have been established and linked to clinical data [8]. Yet, it remains extremely difficult to recover sufficient high-quality RNA from these FFPE samples due to formalin-induced cross-linking and degradation as well as the requirement for large amounts of the FFPE samples and time consuming methods for RNA extraction [9, 10]. Thus, a significant need exists for the development of more efficient methods to recover high-quality nucleic acid material from FFPE samples without sacrificing large amounts of these valuable samples [8].

Members of our team have previously developed disruption and nucleic acid isolation technologies that have proven useful to the biomedical research and clinical communities [11, 12]. We hypothesized that the microHomogenizer™ (mH) device could significantly disrupt FFPE tissue to liberate more high-quality RNA material and increase the efficiency of RNA purification from FFPE samples using standard kits. In the present study, the combination of our mH technology with the Qiagen FFPE RNA extraction kit has significantly improved RNA quality and yield from pancreatic ductal adenocarcinoma (PDAC) patient and cell line derived xenograft FFPE samples. We have focused this new RNA extraction technology on PDAC, since this recalcitrant disease is in need of new biomarkers for early diagnosis [13–15]. We have validated the mH technology on FFPE specimens by demonstrating that gene expression signatures obtained for key biomarker genes agree with those that have been previously published using freshly-frozen specimens [16]. The method further enabled us to evaluate the microenvironmental and KRas mutant effects on biomarker expression in PDAC xenograft mouse models; most importantly, showing very large differences between patient tumors growing at orthotopic (e.g., patient-derived orthotopic xenografts [PDOX]) and subcutaneous sites. Such methodological improvements and translational insights will certainly aid researchers and clinicians in efforts to diagnose and treat PDAC, as well as other malignancies, using NGS technology.

## RESULTS

### The mH increases RNA yield from PDAC FFPE samples

**Figure 1:**
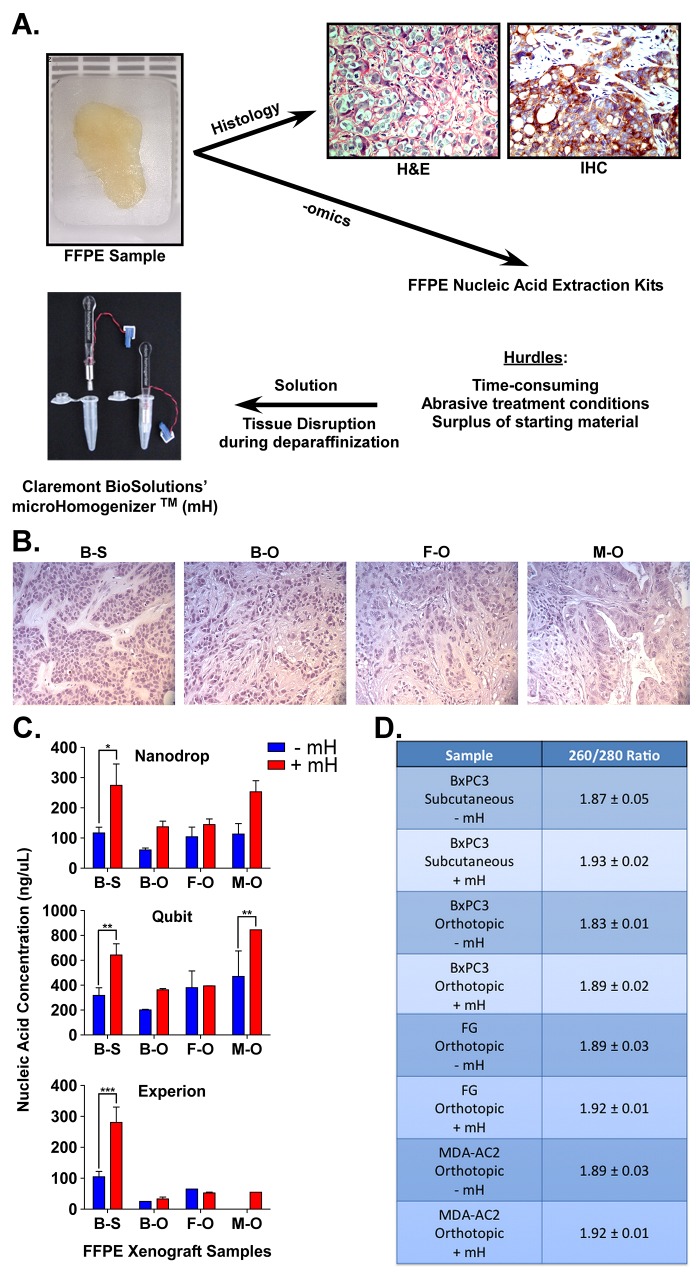
**A**. FFPE samples are usually used for hematoxylin and eosin (H&E) and/or immunohistochemistry (IHC) stains. To overcome hurdles associated with gene expression analyses of tissue in FFPE samples, we show that the mH can increase the purification of high-quality RNA for downstream applications. **B**. H&E staining of the four xenograft FFPE samples (BxPC3 [B] and FG [F]) or patient (MDA-AC2 [M]) xenografts (S = subcutaneous, O = orthotopic). **C**. Nucleic acid (RNA) concentrations (ng/uL) for the four FFPE samples of PDAC using either the standard Qiagen FFPE RNA extraction (-mH) or our mH-modified protocol (+mH). Measurements were made using Nanodrop, Qubit and Experion Bioanalyzer assays. **D**. Average +/- standard error mean (SEM) RNA 260/280 ratios for the same samples described in (A). *, **, *** indicate student t-test p values < 0.05, 0.01 and 0.001, respectively.

**Figure 2:**
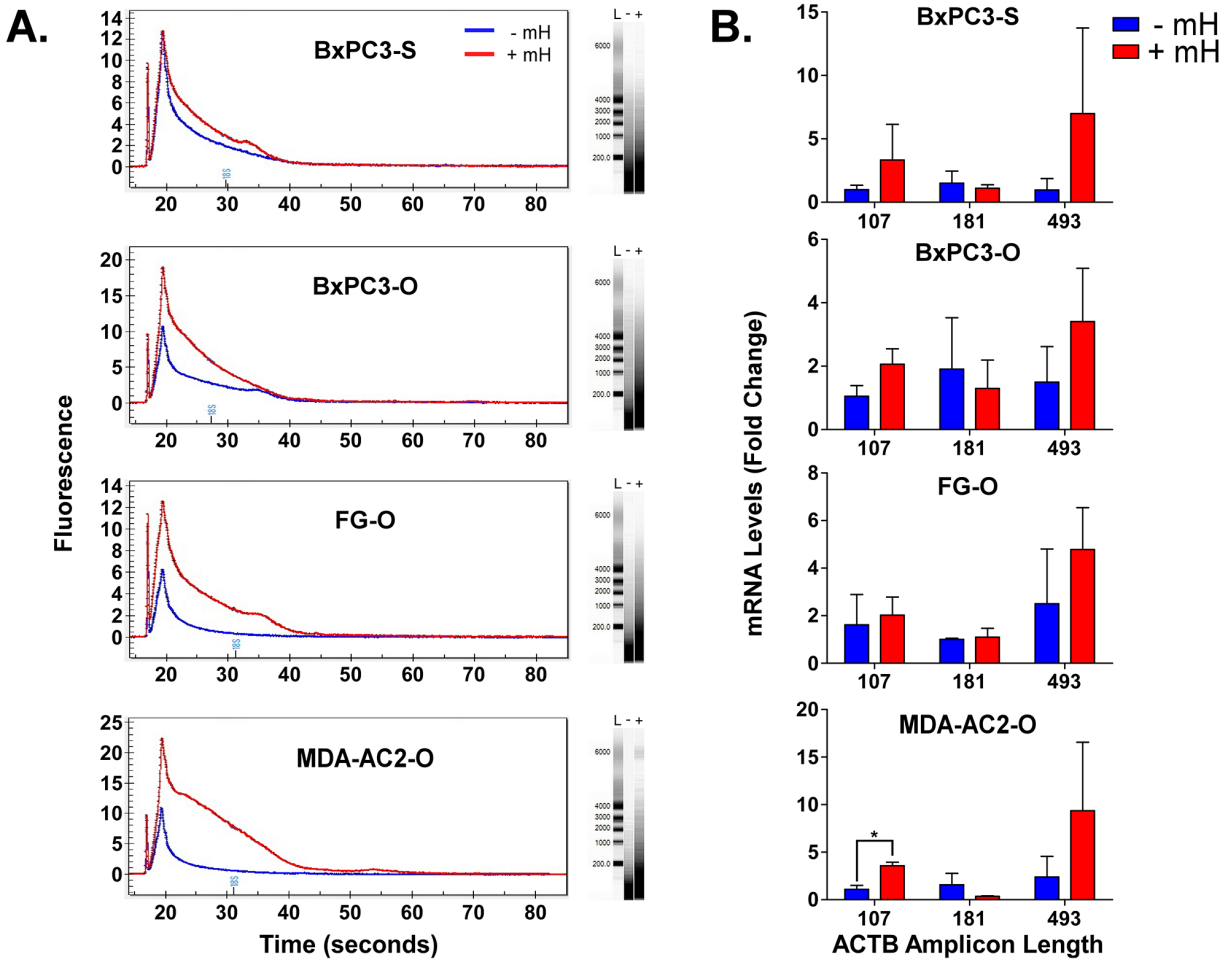
**A**. Overlay of representative Experion-generated electropherograms and gels for total extracted FFPE RNA from either the -mH or +mH RNA extraction methods. L, - and + in the gels indicates lanes for the ladder, -mH and +mH sample preparation methods. **B**. RNA extracted from FFPE tumor samples using these two methods was reverse transcribed to cDNA and analyzed by qPCR in triplicate using βactin-specific primers that generate increasing length amplicons. βactin Ct values were normalized relative to the HPRT1 and POLR2A house-keeping genes and plotted as relative quantification (RQ) fold change compared to the FFPE samples processed without the mH-based method. * indicates a Student's t-test p value < 0.05.

Figure 1A shows an example of formalin-fixed, paraffin-embedded (FFPE) samples used in oncology and pathology for hematoxylin and eosin (H&E) staining as well as immunohistochemistry (IHC). Importantly, FFPE samples also hold great promise for biomarker identification since there is a surplus of these samples linked to clinical data. However, a major hurdle in utilizing these samples for next-generation sequencing (NGS) or other -omics methods is that the RNA material within FFPE samples is commonly degraded and retrieving sufficient high-quality material for such methods is prohibitive. To address this hurdle, we collaborated with Claremont BioSolutions and applied their microHomogenizer^TM^ (mH) device to liberate more intact, high-quality RNA during this standard FFPE purification method. FFPE samples were generated from palpable tumors grown as either subcutaneous or orthotopic xenografts of BxPC3 (KRas wild type PDAC line), FG (KRas mutant PDAC line) or MDA-AC2 (primary patient PDAC tissue) samples in nude (*nu/nu*) mice. Figure 1B shows representative H&E images of each tumor sample indicating the tumor morphology. Notably, orthotopic xenografting yielded tumors with more stromal infiltration, indicative of a more physiologically relevant tumor microenvironment (TME). RNA yield and purity were measured for PDAC FFPE samples processed using the Qiagen FFPE RNA purification kit according the manufacturer recommendations, with or without the mH. While the Qiagen kit is typically used without any sample disruption method, we adapted the method by adding two steps of two-minute sample homogenization using the mH (at 6 volts). Once RNA was extracted from each sample, three different devices were used for its analysis (Figure 1C and 1D). A NanoDrop 2000c was used to quantify both the concentration (ng/uL) and purity (A260/A280 ratio) of RNA extracted (Figure 1C and 1B). A ratio of ~2.0 is generally accepted as “pure” for RNA (Figure 1B). A Qubit and Experion bioanalyzer were also used to quantify RNA concentration (ng/uL) (Figure 2A). Notably, RNA purified using our mH-based method consistently yielded higher concentrations of RNA (Figure 2C) and RNA of higher purity (Figure 1D).

### The mH increases RNA integrity & length from PDAC FFPE samples

A major hurdle in using FFPE samples as an upstream source for NGS-based biomarker research is the poor quality of RNA that results from the extraction process [9, 10]. Since our mH-based method was able to liberate increasing amounts of RNA from the FFPE samples, we further reasoned that the mH-purified RNA may contain longer RNAs of increasing integrity. To test this hypothesis, we analyzed FFPE RNA extracted with and without the mH using an Experion bioanalyzer. As shown in Figure 2A, electropherograms and gel separation of the FFPE RNA purified using the mH-based method contains significantly longer RNA molecules with bands indicating RNA lengths of up to 6000 bases. We further evaluated the quality of these longer RNA molecules using qPCR to detect intracellular β-actin with primer variants designed to produce various amplicon sizes (i.e., 107, 181 and 493 bps). Notably, the mH-purified RNA produces higher β-actin RQ values consistently for the longer amplicon primers (Figure 2B). These results suggest that our improved mH-based FFPE RNA extraction protocol significantly increases RNA yield, purity and integrity.

### mH-mediated RNA purification maintains tumor microenvironment (TME)-specific gene expression profiles

**Figure 3:**
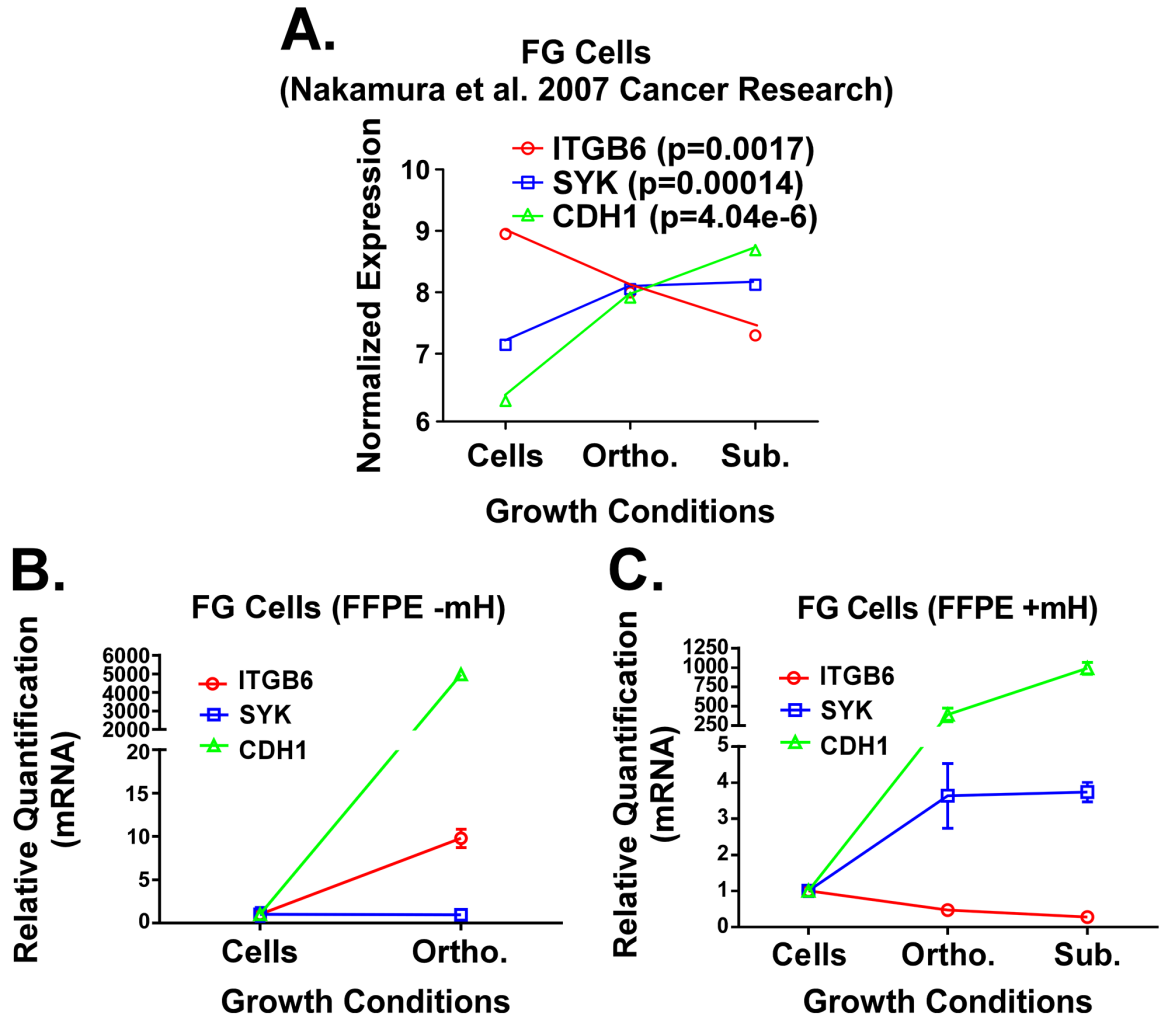
**A**. Previously published expression patterns for ITGB6, SYK and CDH1 genes (Nakamura et al.) in FG cells grown under 2D *in vitro*, subcutaneous *in vivo* and orthotopic *in vivo* microenvironment conditions. RNA was processed from fresh/frozen samples. **B and C**. qPCR analysis for these same three genes in RNA extracts from FG cells grown *in vitro* or FFPE samples of FG cell xenografts. RNA was isolated using the standard Qiagen FFPE RNA kit protocol (B) or our mH-modified protocol (C). Relative quantification (RQ) values for gene expression were normalized to house-keeping genes (GAPDH and/or POLR2A) and calculated relative to gene expression levels in cells grown *in vitro*.

**Figure 4:**
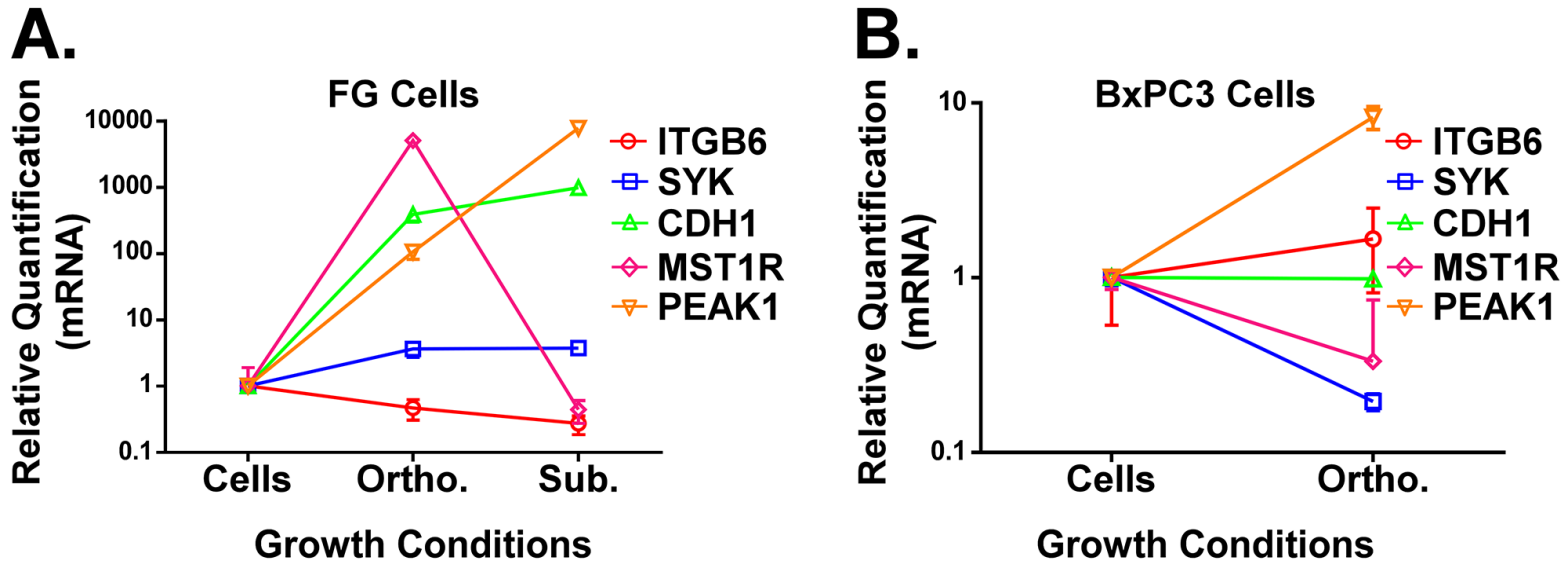
**A and B**. qPCR analysis of ITGB6, SYK, CDH1, PEAK1 and MST1R in FG (A, KRas mutant G12D line) or BxPC3 (B, KRas wild type line) PDAC cells grown under the indicated microenvironmental conditions. All RNA was extracted using our mH-modified Qiagen FFPE kit protocol and relative quantification (RQ) values for gene expression were normalized to house-keeping genes (GAPDH and/or POLR2A).

The importance of the TME in the initiation and progression of cancer is well established [16–19]. We plotted normalized gene expression values reported by Nakamura et al. using fresh-frozen specimens of FG PDAC cells grown *in vitro* or *in vivo* as orthotopic or subcutaneous xenografts (Figure 4A) [16, 19]. For our analyses, we chose to focus on the expression pattern for three genes that were previously reported to be part of a KRas-dependency signature in PDAC and lung cancer [20]. Notably, maximal expression for the SYK and CDH1 genes was observed by Nakamura and colleagues when the FG cells were propagated *in vivo* as subcutaneous xenografts, while they reported maximal expression for ITGB6 when the cells were cultured *in vitro* (Figure 3A). We then harvested RNA from FG cells grown *in vitro* or *in vivo* as orthotopic xenograft tumors using either the Qiagen RNA or FFPE RNA purification kits without the use of the mH. As shown in Figure 3B, the gene expression trend for CDH1 in FG cells grown *in vitro* versus as an orthotopic xenograft matches that from Nakamura and colleagues. However, these trends for both the SYK and ITGB6 genes are opposite to those reported by Nakamura et al. In contrast, when we used our mH-based method to purify the RNA from FFPE samples of FG cells grown *in vivo* as subcutaneous vs. orthotopic tumors, the trends in expression for these three genes match those previously reported by Nakamura et al. using fresh-frozen material from these three microenvironments (Figure 3C). These results, together with the data presented above, demonstrate that using the mH during RNA purification from FFPE tumor samples enables gene expression studies that more faithfully recapitulate the gene expression patterns generated from fresh-frozen tissue.

### Analysis of TME effects on PDAC biomarkers in KRasG12D vs. KRasWT Cells

Not only are activating Ras mutations the most common oncogenic alteration among solid cancers, it is well established that mutations that constitutively activate KRas are an early event in the pathogenesis of nearly all pancreatic cancers [13, 14, 18]. Nonetheless, some PDACs do not contain this oncogenic mutation. Thus, it is relevant to investigate how the microenvironment changes gene expression patterns for key biomarkers in KRas mutant and KRas wild type PDAC models. We harvested RNA from FG (KRasG12D mutant) and BxPC3 (KRas wild type) PDAC cells grown *in vitro* or as *in vivo* xenografts using our mH-based extraction method of RNA from the FFPE tumor samples. We subsequently analyzed gene expression for the genes shown in Figure 3 as well as MST1R and PEAK1. MST1R (or the RON receptor) was previously reported by Singh and colleagues to be part of a KRas dependency gene signature [20] and can promote resistance to the chemotherapy drug gemcitabine [21]. We have previously reported that PEAK1 kinase is essential for the initiation and progression of pancreatic cancer [22–24]. As shown in Figure 4A, with the exception of ITGB6, the expression for these other biomarker genes increases when the KRas mutant FG cells are propagated in the orthotopic pancreatic microenvironment relative to *in vitro*. Notably, PEAK1 and MST1R expression levels were decreased and increased, respectively, by over 100-fold in KRas mutant PDAC cells xenografted into the orthotopic microenvironment relative to the subcutaneous microenvironment (Figure 4A). In contrast, only PEAK1 expression levels increase in the KRas wild type BxPC3 cells when they are grown *in vivo* (Figure 4B).

## DISCUSSION

In conclusion, we have established a new method for purifying RNA from FFPE tumor samples that liberates more high quality RNA for downstream applications. This method has been validated using qPCR to compare gene expression of important PDAC biomarkers previously published from fresh-frozen samples with those generated from our FFPE samples for the same cells and same microenvironments (Figure 3). Importantly, incorporating the Claremont BioSolutions’ mH into commercially available FFPE RNA purification protocols can improve RNA yield, purity and integrity yielding more accurate results in gene expression studies (Figures 1 and 2). Furthermore, we predict that this will have an integral positive impact on future NGS studies, since unlike qPCR or microarray technology, the accuracy of this technology requires much more high quality RNA in the starting material.

Additionally, we have demonstrated that MST1R and PEAK1, two well-established regulators of PDAC progression and therapy resistance [21–25], may fulfill distinct roles during the progression of KRas mutant and KRas wild type PDAC. While PEAK1 is upregulated in both genetic backgrounds within PDAC cells under physiologically relevant tumor microenvironment conditions (i.e., the orthotopic site in nude mice), MST1R is only upregulated in KRas mutant cells implanted at this site (Figure 5). While additional work is needed to assess the functional relevance of both PEAK1 and MST1R in the context of KRas wild type cancers, these data predict that PEAK1 may be a more potent driver of initiation and progression under these wild type KRas conditions. However, while our qPCR primers were designed to detect all gene product isoforms, it will be important for future sequencing studies to evaluate the isoform-specific expression patterns for these genes as isoform differences for the MST1R gene have been implicated in its transformative potential [25]. We predict that this new mH-based RNA extraction method can now be applied to evaluate tumor-specific factors that can regulate the splicing and tumorigenic potential for MST1R and other genes associated with PDAC. Our data further emphasize the importance of studying PDAC as well as other cancer types as patient-derived orthotopic xenografts (PDOX) rather than as subcutaneous xenografts in nude mouse models [26–35].

Previously developed concepts and strategies of highly selective tumor targeting can now take advantage of these new methods of biomarker measurement using the mH-based RNA extraction method described here and NGS technology to identify differences between the microenvironment of normal and tumor tissue [36–41].

## MATERIALS AND METHODS

### Cell culture

BxPC3 and FG cells were cultured in complete Dulbecco's Modified Eagles Medium (DMEM) supplemented with 10% fetal bovine serum and antibiotics. Cells were maintained at 5% CO_2_ and 37°C.

### Animal care

Athymic nu/nu nude mice between 4 and 6 weeks of age were maintained in a barrier facility on high efficiency particulate air (HEPA)-filtered racks. The animals were fed with autoclaved laboratory rodent diet (Teckland LM-485; Western Research Products, Orange, CA). All animal studies were approved by the UCSD Institutional Animal Care and Use Committee and conducted in accordance with the principles and procedures outlined in the NIH Guide for the Care and Use of Animals.

### Sourcing of human tumor tissue

The tumor tissue from the MDA-AC2 sample was obtained and initially xenografted subcutaneously at the M.D. Andersen Cancer Center [29–35] in accordance with Institutional Review Board (IRB) approval. Tissue was obtained at the time of tumor resection. Excess fresh tumor was used for immediate xenografting into mice. All surgically-resected tumor fragments were stored in sterile specimen cups and expeditiously transported from the operating room to the laboratory on ice.

### Xenografting

MDA-AC-2 tumors growing subcutaneously were harvested and implanted orthotopically at AntiCancer, Inc., in accordance with the principles and procedures outlined in the NIH Guide for the Care and Use of Animals under Assurance number A3873-01. Alternatively, PDAC cell cultures were prepared at 1×10^6^ cells/20 μl for antibiotic-free DMEM for implantation. *Nu/nu* mice were anesthetized with intraperitoneal injection of ketamine xylazine cocktail (60 mg/kg: 10 mg/kg). They were then placed in the supine position on a warm pad to maintain body temperature. Once mice were sedated, the abdominal wall was cleansed with 70 alcohol and betadine. For orthotopic xenografting, a 1-2 cm midline incision was made in the left lateral flank through the skin, fascia, and peritoneum, and the tail of the pancreas was exposed. For patient-derived tissue fragments, surgical sutures (6-0 silk) were used to implant tumor fragments (1 mm^3^) onto the pancreas [26, 27]. For PDAC cell suspensions, 20 μl was injected directly into the pancreas. The pancreas was then returned to the abdomen, and the peritoneum and skin were closed using 6-0 Polysorb surgical suture. For subcutaneous xenografting, 100 μl of the cell suspension was injected into each flank of the mice. Mice were monitored daily for 5 consecutive days after surgery with particular attention paid to animal distress, wound dehiscence, and signs of infection. Thereafter, they were examined daily. Tumor progression was also evaluated by ultrasound every 3-4 weeks. Animals were euthanized based on either tumor volume (threshold 2500 mm^3^) as determined by ultrasound or clinical status during the observation period as specified in our IACUC-approved protocol. Tumors were harvested and fixed in 10% formalin for 16 hours prior to exchanging the formalin for 95% ethanol and subsequent processing and analysis.

### Sample processing

Formalin-fixed tissue specimens were processed for paraffin embedding and sectioning at the UCLA pathology core and returned to the research team as paraffin blocks, H&E stained sections and unstained sections.

### RNA extraction

FFPE samples were weighed out to approximately 10mg each and then the Qiagen FFPE RNA extraction kit was used either with or without the Claremont Biosolutions’ microHomogenizer™ (mH) device. For mH-modified protocol, the microHomogenizer was used twice during the deparaffinization step for 2-minute cycles at power of 6 volts. The remainder of the protocol was followed as directed by the kit instruction manual. RNA harvested from cells propagated *in vitro* was purified using the standard Qiagen RNA purification kit.

### RNA quantification

RNA concentrations were quantified using three methods: NanoDrop, Qubit, and Experion. For NanoDrop runs, the instrument was blanked with nuclease-free water and samples were analyzed to determine nucleic acid concentrations in ng/uL as well as 260/280 ratios. For Qubit runs, the instrument was calibrated using Qubit® RNA Broad-Range assay kit by Life Technologies. Buffer (198 μL) was used with 1 μL of dye and 1 μL of sample. Samples were analyzed to determine nucleic acid concentrations in ng/μL. For Experion runs, the Experion™ RNA StdSens Analysis Kit by Bio-Rad Laboratories was used. RNA StdSens Chip was prepared according to the manufaturer's protocol.

### Reverse transcription and qPCR

The Fermentas Maxima Universal Strand cDNA kit was used to synthesize cDNA using 100ng of template and both oligo(dT) and random hexamer primers according the manufacturer's protocol. cDNA concentrations were determined by NanoDrop and then diluted to 22.5ng/mL to perform qPCR. Primers were purchased from Intergrated DNA technologies and used at a concentration of 10nmol/mL. 8.75μL of nuclease-free water was mixed with 2.5μL of diluted cDNA, 1.25μL of gene-specific primer, and 12.5μL of Thermo Scientific Maxima SYBR Green/ROX qPCR Master Mix. Samples were run on an ABI 7300 instrument. Average ΔΔCT values from three independent qPCR experiments were used to calculate average relative quantification units and plotted as fold change relative to the appropriate control.

### PCR primers

ACTB-493 – AGCCATGTACGTTGCTATCC/GTACAGGTCTTTGCGGATGT

ACTB-181 – GGAAATCGTGCGTGACATTAAG/GAAGGAAGGCTGGAAGAGTG

ACTB-107 – GGACCTGACTGACTACCTCAT/CGTAGCACAGCTTCTCCTTAAT

CDH1 – GTCACTTCAGACTCCAGCCC/AAATTCACTCTGCCCAGGACG

ITGB6 – CAAGTTGAGTCCTTCAGTGTCT/GACTCCGGAAACATTCTCCAG

MST1R – GTCTGCCTCCCAGCATTG/TTGACCCTTTTGACCTTACCC

SYK – TCTTGTCTTTGTCGATGCGAT/CTCGGGAAGAATCTGAGCAAA

PEAK1 – GCTGAGATTCTTTAGCTTTGCTC/GACTTCAGGCTAACCAGTGAC

HPRT1 – GCGATGTCAATAGGACTCCAG/TTGTTGTAGGATATGCCCTTGA

POLAR2A – TCGTCTCTGGGTATTTGATGC/CAGTTCGGAGTCCTGAGTC

GAPDH – TGTAGTTGAGGTCAATGAAGGG/ACATCGCTCAGACACCATG

### Statistical analysis

All quantified data were plotted and analyzed in GraphPad Prism 5.0 with using a Student's t-test. Data are representative of at least 3 independent experiments and are reported as replicate averages ± SEM, unless otherwise indicated.
